# From aid dependence to domestic ownership: Risks, challenges, and policy pathways for sustaining immunisation gains in lower-income countries

**DOI:** 10.1016/j.pmedr.2026.103560

**Published:** 2026-07-08

**Authors:** Abdirahman Mohamed Adan, Ahmed Mohamed Omar, Mohamed Sharif Abdi, Maryama Ahmed Ali, Yakub Burhan Abdullahi

**Affiliations:** a Faculty of Health Sciences and Tropical Medicine, Somali National University, Mogadishu, Somalia; b Center for Health Research and Innovation, Somali National University, Mogadishu, Somalia; c Faculty of Health Science, Salaam University, Mogadishu, Somalia

## Abstract

****Objective**:**

Lower-income countries are increasingly expected to finance immunisation programmes as global health financing shifts from donor support to domestic ownership, in line with Immunization Agenda 2030 (IA2030). While rising domestic investment signals progress, concerns remain regarding whether countries have sufficient fiscal space and institutional capacity to sustain immunization gains independently.

****Methods**:**

This commentary synthesizes published evidence from Gavi-supported and transitioning lower-income countries using literature available through April 2026 to evaluate financial, economic, and health system factors influencing the sustainability of immunization programmes.

****Results**:**

Three interlocking risks are identified: limited fiscal space, rising vaccine and delivery costs, and macroeconomic instability linked to overlapping aid transitions. Persistent institutional weaknesses in procurement, budgeting, regulatory systems, and supply chain management further threaten sustainability. Evidence from past transitions shows mixed outcomes, with success associated with early financial planning, strong prioritisation of immunisation, and integration into national health systems.

****Conclusions**:**

Although increased domestic investment is essential for country ownership, many lower-income countries are not yet fully prepared to sustain immunisation gains independently. A phased, risk-sensitive transition combining strengthened domestic financing with continued targeted global support is necessary to prevent reversals in coverage and equity.

Lower-income countries investing a record US$1.1 billion of their own resources in immunisation in 2026 marks a symbolic turning point in global health financing, as highlighted by Gavi's 15 April 2026 announcement of unprecedented domestic commitments to vaccine programmes (Gavi, the Vaccine Alliance, n.d.). This shift from aid dependence to domestic ownership aligns with the Immunisation Agenda 2030 vision of country-led, sustainable immunisation, raises critical questions about whether lower-income countries are truly ready fiscally and institutionally.

Historically, Gavi's model has enabled rapid introduction of new and underused vaccines in over 70 countries, coupled with a phased “co-financing and transition” policy that triggers increased domestic contributions as gross national income rises, culminating in full self-financing after graduation (Kallenberg et al., 2016). This approach has demonstrably expanded coverage and averted millions of vaccine-preventable deaths, while explicitly aiming to hard-wire financial sustainability into programme design (Perera et al., 2024; Huffstetler et al., 2022). Yet donor transitions have been shown to affect every health system “building block,” with poorly managed exits linked to stock-outs, service disruptions, and human resource gaps, underscoring the fragility of gains in many settings (Kolesar et al., 2023).

Evidence from Gavi-graduated and transitioning countries suggests that successful domestic ownership is possible but far from guaranteed. Synthetic control analyses of post-transition performance reveal substantial heterogeneity: some countries maintain or exceed expected coverage, while others experience precipitous declines, especially when broader fiscal and political risks are not adequately factored into transition planning (Huffstetler et al., 2022). Key drivers of successful transitions include early and sustained financial planning, strong political prioritisation of immunisation, robust procurement systems, and effective integration of immunisation into national health system structures. Conversely, transitions have faltered where fiscal space was severely constrained, institutional capacity was underdeveloped, or planning was initiated too close to the graduation date. Case studies from Sri Lanka's Expanded Programme on Immunisation illustrate one pathway to sustained coverage after Gavi exit, achieved through co-financing paired with early financial planning, strong procurement systems, and integration into routine services, enabling domestic resources to fully replace Gavi funds (Saxenian et al., 2015). It should be noted, however, that Sri Lanka represents a relatively strong institutional and fiscal context, and its experience may not be directly transferable to countries with weaker health systems or more constrained fiscal environments. Conversely, mixed performance in other health programmes within the same system illustrates how non-operational components such as training and research are especially vulnerable once donor funding ends (Saxenian et al., 2015).

Three interlocking risks threaten the sustainability of immunisation gains,as illustrated in Fig. 1. First, limited fiscal space: global analyses of development assistance for health project a protracted period of declining aid volumes to 2030, implying that domestic fiscal space, not external replenishments, will increasingly determine immunisation trajectories. Systematic reviews of fiscal space for health in low- and middle-income countries show that macroeconomic growth, tax policy, budget reprioritisation, and efficiency gains are the main levers, but their realistic yield is constrained in many aid-dependent settings (Sriudomporn et al., 2023). Second, rising vaccine and delivery costs: projections for 94 low- and middle-income countries estimate a US$38.4 billion funding gap for 16 vaccines between 2011 and 2030, driven predominantly by delivery costs, with domestic financing increases unable to fully offset anticipated reductions in Gavi and other aid (Cernuschi et al., 2018). Third, economic instability and simultaneous aid transitions: analyses of donor behaviour show that Gavi graduation often coincides with reductions in bilateral health aid, which may increase the risk of funding gaps, particularly for countries with limited geopolitical leverage and constrained domestic fiscal capacity (Ikilezi et al., 2020). These three risks are mutually reinforcing: constrained fiscal space limits a country's ability to absorb rising vaccine and delivery costs, while simultaneous reductions in bilateral aid compound financial pressure precisely at the moment of graduation, when countries are most vulnerable. Together, these dynamics can trigger supply chain disruptions, workforce gaps, and ultimately declines in immunisation coverage and equity.

**Fig. 1:**
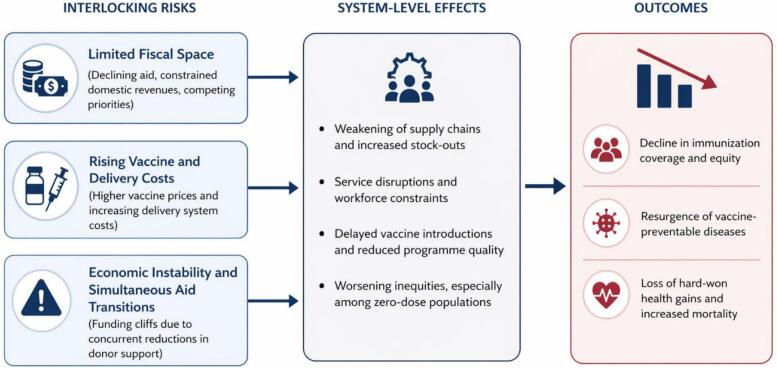
Interlocking risks and pathways threatening the sustainability of immunization gains during donor transition.

Institutional readiness is equally uneven. Reviews of Gavi transitions highlight recurring weaknesses in budgeting, procurement, regulatory capacity, and technical expertise for vaccine planning, even among countries nearing graduation [10]While Gavi's evolving transition and co-financing policy, extending accelerated transition periods and incorporating risk-based flexibility, reflects meaningful learning from early experiences and encompasses both financial and programmatic dimensions (Huffstetler et al., 2022; Jha et al., 2024), assessments suggest that financial indicators continue to receive comparatively greater emphasis in practice, with programmatic sustainability, governance, and supply chain security receiving less systematic attention during transition planning.

Policy responses must therefore balance ambition for domestic ownership with pragmatic risk management. Sustainable financing strategies should combine increased domestic revenues, higher priority for primary health care and immunisation within budgets, and efficiency gains, while recognising that many lower-income countries will require continued, though reconfigured, global support (Sriudomporn et al., 2023). Concretely, Ministries of Finance and Health should develop joint multi-year immunisation financing plans at least five years before Gavi graduation, with pre-agreed domestic budget lines indexed to transition timelines and reviewed annually. Gradual, context-sensitive transition trajectories, allowing longer timelines where fiscal space is constrained and explicitly linked to performance and risk assessments, are preferable to rigid income thresholds (Kallenberg et al., 2016; Huffstetler et al., 2022; Jha et al., 2024). Additionally, Gavi and partner governments should establish country-specific readiness thresholds encompassing procurement capacity, supply chain functionality, and regulatory benchmarks that must be demonstrably met before graduation is confirmed. At the same time, external partners should maintain targeted technical and catalytic financial support post-transition, particularly for high-cost new vaccines and for safeguarding delivery systems serving zero-dose and under-immunised populations (Huffstetler et al., 2022). Post-transition technical support should be ring-fenced and coordinated through WHO country offices to avoid duplication and coverage gaps.

In conclusion, increased domestic investment in immunisation is a necessary and welcome step toward country ownership, but current evidence indicates that many lower-income countries are not yet fully prepared financially or institutionally to sustain gains without carefully managed, risk-responsive transitions. A balanced approach that couples strengthened domestic responsibility with continued, strategically focused global solidarity is essential to prevent backsliding and to secure the hard-won achievements of the past two decades.

## CRediT authorship contribution statement

**Abdirahman Mohamed Adan:** Writing – original draft. **Ahmed Mohamed Omar:** Conceptualization. **Mohamed Sharif Abdi:** Conceptualization. **Maryama Ahmed Ali:** Investigation. **Yakub Burhan Abdullahi:** Supervision.

## Ethical approval

This study did not involve human participants or animals; therefore, ethical approval was not required.

## Declaration of competing interest

The authors declare that they have no known competing financial interests or personal relationships that could have appeared to influence the work reported in this paper.

## Data Availability

No data was used for the research described in the article.
